# System Xc-pathway as a potential regulatory target in neurological disorders

**DOI:** 10.3389/fphar.2025.1701320

**Published:** 2026-01-02

**Authors:** Ying Chen, Wei Xiao, Chengli Qian, Linshu Huang, Jianghao Lv, Zhuo Wang, Yi Luo

**Affiliations:** 1 Department of Laboratory Medicine, Zhongnan Hospital of Wuhan University, Wuhan, Hubei, China; 2 Department of Nephrology, The Central Hospital of Wuhan, Tongji Medical College, Huazhong University of Science and Technology, Wuhan, Hubei, China; 3 Hubei Polytechnic Institute, School of Medical Science, Xiaogan, Hubei, China; 4 Medical Science Research Center, Zhongnan Hospital of Wuhan University, Wuhan, Hubei, China; 5 Department of Neurology, Zhongnan Hospital of Wuhan University, Wuhan, Hubei, China; 6 Hubei Provincial Clinical Research Center for Molecular Diagnostics, Wuhan, Hubei, China

**Keywords:** central nervous system, ferroptosis, glutamate excitotoxicity, NMDARs, oxidative stress, system Xc-

## Abstract

The System Xc-pathway is composed of the 12-transmembrane transporter protein SLC7A11 (xCT) and the single-channel transmembrane protein SLC3A2 (CD98hc). We detail the pathway’s characteristics and distribution within the central nervous system, as well as its canonical role in maintaining glutathione synthesis and inhibiting ferroptosis, and its emerging non-canonical functions in metabolic coupling and neuroimmunity. A core theme is the pathway’s context-dependent and often paradoxical role across major neurological disorders, including ischemic stroke, Alzheimer’s disease, multiple sclerosis, Parkinson’s disease, and amyotrophic lateral sclerosis. Critically, we analyze how pathological activation of N-methyl-D-aspartate receptors (NMDARs) can dysregulate System Xc-through mechanisms involving calcium overload, reactive oxygen species, and specific signaling axes (e.g., Nrf2, PP2A/AMPK/HMGB1), thereby exacerbating excitotoxicity and oxidative damage. Conversely, System Xc-dysfunction can further fuel NMDAR-mediated injury, creating vicious pathogenic cycles. This analysis reveals that System Xc-is not a unitary target but a dynamic node within a complex network. Consequently, effective therapeutic strategies must move beyond broad inhibition and instead aim for nuanced, cell-type-specific, and disease-stage-precise modulation. This approach will selectively correct the dysfunction of System Xc-while preserving its essential physiological roles. It presents both a significant challenge and a promising frontier for future neuroprotective drug development.

## 1 Introduction

The central nervous system (CNS) maintains a delicate balance between excitatory signaling and antioxidant defense. Disruption of this equilibrium underpins a wide spectrum of neurological disorders. The System Xc-pathway, a cystine/glutamate antiporter, sits at this critical nexus, orchestrating both synaptic glutamate homeostasis and the biosynthesis of glutathione (GSH), the master cellular antioxidant (Lin et al., 2020). Its dysfunction is mechanistically linked to two pivotal forms of neuronal death: excitotoxicity, driven by excessive glutamate receptor activation, and ferroptosis-an iron-dependent, lipid peroxidation-driven cell death process (Sontheimer, 2008; Dixon et al., 2012; Shi et al., 2023). Consequently, System Xc-dysregulation is implicated in the pathogenesis of various CNS disorders, including ischemic brain injury, Alzheimer’s disease (AD), multiple sclerosis (MS), Parkinson’s disease (PD), amyotrophic lateral sclerosis (ALS), and other iron death-related diseases (Bridges et al., 2012; Conrad and Sato, 2012; Lewerenz et al., 2013).

Previous studies have suggested that glutamate accumulation serves as the initiator of neuroexcitotoxicity and ferroptosis, primarily through overstimulation of glutamate receptors, specifically N-methyl-D-aspartate receptors (NMDARs), while implicating the inhibition of System Xc-in the non-receptor-mediated pathway (DeGregorio-Rocasolano et al., 2019; Fan et al., 2023). However, emerging evidence suggested that NMDARs-mediated signaling may also contribute to the crosstalk between excitotoxicity and ferroptosis, potentially through mechanisms that are independent of glutamate stimulation (Han et al., 2024; Fan et al., 2025). Here, we systematically review the role of System Xc-pathway in neurological disorders, with a particular focus on the potential regulation via NMDARs-mediated signaling, aiming to provide a theoretical basis for the development of neuroprotective strategies targeting System Xc-to modulate oxidative defense and glutamate homeostasis in the CNS.

## 2 System Xc-pathway in central nervous system

### 2.1 The characteristics and distribution of system Xc-in CNS

System Xc-is an obligate heterodimeric antiporter composed of the specific light chain subunit SLC7A11 (xCT) and the heavy chain subunit SLC3A2 (4F2hc/CD98), linked via a disulfide bond (Lin et al., 2020; Conrad and Sato, 2012). SLC7A11, possessing 12 transmembrane domains, confers substrate specificity, mediating the 1:1 electrogenic exchange of extracellular cystine for intracellular glutamate. SLC3A2, a single-pass transmembrane protein, is essential for the correct plasma membrane trafficking and stabilization of the complex. Previous studies have shown that System Xc-is broadly expressed across various cell types within the CNS, including neurons, astrocytes, microglia, and oligodendrocytes, underscoring its significant and versatile role in the physiological and pathological processes of the CNS (Burdo et al., 2006; Yi et al., 2023) (Table 1).

**TABLE 1:** System Xc-in CNS cell types: from physiological expression to pathological dysregulation.

Cell types	Physiological condition	Pathological condition
Neurons	Low expression of System Xc- in cortical neurons but high in CA1 pyramidal neurons in the hippocampus (Lewerenz et al., 2013) SLC7A11 protein barely detectable in mature GABAergic (inhibitory) neurons (Han et al., 2024) System Xc- highly expressed in certain neuronal subtypes, such as glutamatergic neurons (Burdo et al., 2006)	SLC7A11 expression is activated by Nrf2 pathway under oxidative stress (Fan et al., 2025) SLC7A11 is upregulated in response to glutamate toxicity or inflammation (Lewerenz et al., 2012) Ischemia/reperfusion increases p53 activity, which binds to the SLC7A11 promoter and represses its transcription in neurons (Chen et al., 2021)
Astrocytes	Astrocytes express high levels of System Xc- and are the most abundant SLC7A11-expressing cells in the CNS (Jackman et al., 2012) SLC7A11 particularly concentrated in high-density clusters on astrocytic peduncle membranes (Ottestad‐Hansen et al., 2018)	Under hypoxic conditions, astrocytes exhibit enhanced System Xc- activity, which increases glutamate release and contributes to excitotoxicity (Jackman et al., 2012) IL-1β upregulates SLC7A11 expression in astrocytes (Shi et al., 2016) *Slc7a11* mRNA and protein levels are elevated in astrocytes in epilepsy models (Zhang et al., 2024; Alcoreza et al., 2021)
Microglia	SLC7A11 nearly undetectable in resting microglia (Ottestad‐Hansen et al., 2018) Low levels of System Xc- regulate the redox state and immune response in microglia (Beckers et al., 2024)	Microglia upregulate SLC7A11 expression and release more glutamate in response to inflammatory stimuli (Beckers et al., 2024) LPS induction increases SLC7A11 expression in microglia, worsening neuroinflammation (Kitagawa et al., 2019) SLC7A11 is upregulated only in activated microglia and serves as a direct source of glutamate excitotoxicity *in vivo* (Kigerl et al., 2012)
Oligodendrocytes	SLC7A11 moderately expressed in oligodendrocytes and crucial for protecting white matter from oxidative damage (Lewerenz et al., 2013; Yi et al., 2023) During the myelin formation phase, SLC7A11 expression is higher in oligodendrocytes, likely due to increased metabolic demands (Soria et al., 2016)	System Xc- expression is upregulated in oligodendrocytes under oxidative stress and inflammatory conditions (Lewerenz et al., 2013) In ALS, SLC7A11 expression in mature oligodendrocytes is reduced, leading to axonal demyelination and excitotoxic death of motorneurons (Philips et al., 2013)

System Xc-subunits SLC7A11 and SLC3A2 are predominantly detected in neurons of both mouse and human brains, with notable enrichment in immature cortical neurons, where they support synapse development and neurotransmission (Lewerenz et al., 2013; Burdo et al., 2006). Under normal conditions, neuronal SLC7A11 expression is relatively low but can be significantly upregulated in response to glutamate-induced oxidative toxicity or inflammatory stimuli, reflecting an adaptive mechanism to counteract stress (Lewerenz et al., 2012). This activity furnishes the cysteine necessary for GSH synthesis and contributes to the regulation of the extracellular glutamate pool, thereby playing a crucial in maintaining glutamate homeostasis, synaptic plasticity, and overall neuronal function (Jackman et al., 2012).

In astrocytes, System Xc-serves as a major non-vesicular glutamate exporter, critically influencing glutamate homeostasis and excitotoxic thresholds (Dahlmanns et al., 2023). Microglia, the resident immune cells of the CNS, also express System Xc-, which aids in regulating redox balance and immune responses. Upon activation by pro-inflammatory signals such as TNF-α and LPS, microglia markedly upregulates SLC7A11, underscoring a direct link between System Xc-activity and neuroinflammatory processes (Beckers et al., 2024). Within oligodendrocytes, SLC7A11 is moderately expressed and is essential for protecting white matter from oxidative damage (Bridges et al., 2012; Lewerenz et al., 2013). Its expression is observed in both precursor and mature oligodendrocytes, and its dysfunction in these cells is implicated in demyelinating diseases such as multiple sclerosis (MS), emphasizing its importance in maintaining the integrity of myelin and overall white health (Lewerenz et al., 2013; Yi et al., 2023).

Furthermore, System Xc-components are highly expressed at key interfaces between the brain parenchyma and the periphery, including the endothelial cells of the blood-brain barrier (BBB), ventricular cells, and the choroid plexus. This strategic localization suggests its involvement in modulating BBB permeability and function, thereby influencing the exchange of substances between the CNS and peripheral circulation - a role particularly pertinent in neuroinflammatory and neurodegenerative contexts where BBB integrity is compromised (Bridges et al., 2012).

### 2.2 System Xc- and glutamate homeostasis

Glutamate homeostasis in the brain is maintained by diverse transporter proteins. These include intracellular vesicular transporters that package glutamate and plasma membrane transporters that mediate its flux (Mahmoud et al., 2019; Todd and Hardingham, 2020). Among them, System Xc-, a sodium-independent exchanger highly expressed in astrocytes, plays a key role (Bridges et al., 2012). It imports extracellular cystine in exchange for intracellular glutamate at a 1:1 stoichiometry, serving a major source of non-vesicular, extrasynaptic glutamate release (Lin et al., 2020; Bridges et al., 2012). This release modulates extrasynaptic glutamate receptors, including NMDARs, thereby influencing excitotoxicity thresholds and behavior (Massie et al., 2015).

In contrast, glutamate clearance is primarily mediated by the sodium-dependent excitatory amino acid transporters (EAATs), particularly EAAT1 and EAAT2 on astrocytes, which uptake glutamate to prevent excitotoxicity (Todd and Hardingham, 2020; Vandenberg and Ryan, 2013). System Xc-is co-expressed with EAAT1 in astrocytes, and the two systems exhibit a dynamic, often reciprocal relationship (Burdo et al., 2006; Dahlmanns et al., 2023). For instance, aging in rodents is associated with increased expression of System Xc-subunits but decreased levels of astrocytic EAATs (Todd and Hardingham, 2020; La Bella et al., 2007; Souza et al., 2015; Zhang et al., 2016). Extracellular glutamate can rise through either System Xc-activation or EAAT1/2 inhibition, and System Xc-deletion can upregulate EAAT2 during seizures (Dahlmanns et al., 2023; Simón-Sánchez et al., 2025; Loewen et al., 2019). This interplay suggests a compensatory regulatory network that tightly controls extracellular glutamate levels. Thus, understanding glutamate homeostasis requires simultaneous examination of both System Xc- and EAAT1/2 at the cellular level.

### 2.3 System Xc- and oxidative defense

Neurons in the CNS are highly vulnerable to oxidative stress owing to their high oxygen demand and lipid-rich environment, necessitating robust antioxidative defenses (Salim, 2017; Wang and Michaelis, 2010). System Xc-serves as a key pathway involved in this response (Albrecht et al., 2010). In non-receptor-mediated excitotoxicity, elevated extracellular glutamate inhibits System Xc-, resulting in GSH depletion, accumulation of reactive oxygen species (ROS), lipid peroxidation, and ultimately neuronal death (Fan et al., 2023; Robert et al., 2015). The sustained upregulation of System Xc-observed in aging rodents animals may reflect an adaptive mechanism to counteract rising oxidative stress (Souza et al., 2015). The balance between ROS-induced lipid peroxide generation and clearance is essential for preventing ferroptosis, a process critically regulated by the System Xc-/GSH/GPX4 axis (Imai et al., 2017). These mechanisms also inform therapeutic strategies. In neuro-oncology, System Xc-inhibitors, such as sulfasalazine (SAS) can promote glioma cell death (Chen et al., 2015; Sehm et al., 2016). Conversely, in non-neoplastic CNS disorders, enhancing System Xc-activity - through SLC7A11 activation, overexpression, or epigenetic modulation - offers a promising approach to fortify antioxidant defenses and suppress ferroptosis (Li et al., 2023; Costa et al., 2023).

### 2.4 Emerging roles of system Xc-in metabolism and immunity

While System Xc-is canonically known for supplying cystine for GSH synthesis and inhibiting ferroptosis, recent evidence underscores its critical ferroptosis-independent functions with significant pharmacological implications. Beyond antioxidant defense, glutamate exported via System Xc-can be utilized by neighboring cells to generate α-ketoglutarate for the TCA cycle, suggesting a role in modulating neuron-astrocyte metabolic coupling and cellular bioenergetics, requiring further exploration (Zhu et al., 2024). Furthermore, System Xc-activity influences the redox tone, which regulates NLRP3 inflammasome activation and cytokine profiles (e.g., interleukin-1β release) in macrophages during liver fibrosis. This immunomodulatory function appears distinct from its role in preventing lipid peroxidation (Kim et al., 2021). Given that microglia are the resident macrophages of the CNS, the potential immunomodulatory role of System Xc-in microglia warrants careful consideration. Additionally, emerging reports indicate that mTOR-dependent upregulation or phosphorylation of SLC7A11 links altered growth factor receptor signaling with amino acid metabolism and ROS buffering in tumor cells, an insight equally pertinent to CNS pathophysiology (Li et al., 2019; Gu et al., 2017). Therefore, viewing System Xc-merely as an “antioxidant transporter” is inadequate. Pharmacological inhibitors like sulfasalazine or erastin may exert complex effects on neuroimmune crosstalk and brain metabolism, contributing to potential off-target outcomes observed in preclinical models.

## 3 The potential impact of NMDARs-mediated signaling on the system Xc-pathway

**FIGURE 1:**
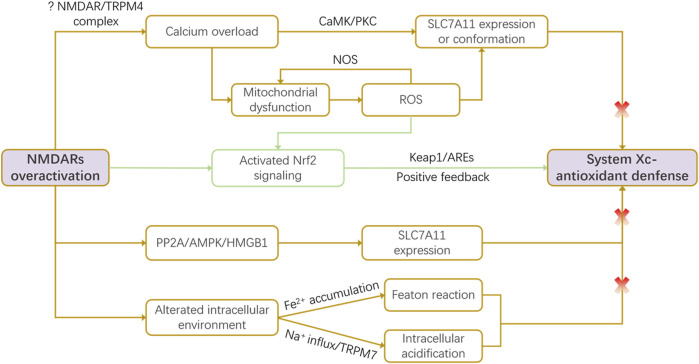
NMDAR-mediated dysregulation of the System Xc-pathway. Under pathological conditions, overactivation of N-methyl-D-aspartate receptors (NMDARs) impairs System Xc-function through multiple convergent mechanisms: calcium overload-induced reactive oxygen species (ROS) production, activation of the PP2A/AMPK/HMGB1 signaling axis, and detrimental alterations in the intracellular milieu (e.g., iron accumulation, acidification). Conversely, NMDAR/ROS signaling can also trigger a compensatory feedback mechanism via Nrf2 activation, which upregulates *slc7a11* transcription in an attempt to restore redox balance. This intricate crosstalk positions System Xc-as a critical node linking excitotoxicity to oxidative stress.

Glutamate activates various receptors, with the ionotropic NMDAR being pivotal for synaptic plasticity, learning, memory, and excitotoxicity (Fan et al., 2023; Hansen et al., 2021). Notably, glutamate serves as a critical link between NMDAR-mediated excitotoxicity and mitochondrial dysfunction across diverse neuropathologies, including neurodegenerative diseases and acute conditions like stroke (Mira and Cerpa, 2021). This interplay often manifests as a vicious cycle involving NMDAR overactivation, oxidative stress, and impaired mitochondrial dynamics (Nguyen et al., 2011). System Xc-, a key regulator of glutamate homeostasis and oxidative defense, has been shown to interact intricately with NMDARs-mediated excitotoxicity in recent studies (Fan et al., 2023; Nakano et al., 2019; Okada et al., 2019). During cerebral ischemia and hypoxia, disrupted ionic gradients lead to membrane depolarization and excessive synaptic glutamate accumulation. This overactivates NMDARs, causing pathological calcium influx (Fan et al., 2023; Yu et al., 2023). Concurrently, energy failure and hypoxia induce oxidative stress, which can stimulate System Xc-to export more glutamate. This creates a positive feedback loop: NMDAR-driven excitotoxicity promotes System Xc-to mediate glutamate release, which further exacerbates NMDAR activation, amplifying cellular damage (Fan et al., 2023). Further, impaired System Xc-function depletes GSH, weakening antioxidant defenses and increasing neuronal vulnerability to NMDAR-mediated oxidative injury. Critically, NMDAR overactivation itself can impair System Xc-function through multiple downstream mechanisms, detailed below (Conrad and Sato, 2012; Fan et al., 2023) (Figure 1).

### 3.1 Mechanisms of NMDAR-mediated system xc-dysregulation

#### 3.1.1 Calcium overload-induced ROS

NMDAR overactivation leads to a massive Ca^2+^ influx, a primary driver of excitotoxic damage. This calcium surge can impair System Xc-function through both signaling and direct oxidative mechanisms. Elevated intracellular Ca^2+^ activates downstream kinases such as calmodulin-dependent protein kinase (CaMK) and protein kinase C (PKC), which may regulate the expression or activity of the System Xc-subunit SLC7A11, influencing cell survival (Han et al., 2024; Ye et al., 2022). More directly, Ca^2+^ overload triggers mitochondrial dysfunction and endoplasmic reticulum stress, promoting the opening of the mitochondrial permeability transition pore (mPTP) and generating excessive ROS (Han et al., 2022; Dixon et al., 2014). High levels of ROS can oxidize critical cysteine residues on the SLC7A11 protein, altering its conformation and inhibiting its cystine/glutamate exchange activity. This results in diminished cystine uptake and GSH depletion, creating a feed-forward loop where excitotoxicity disables a key antioxidant pathway (Han et al., 2024; Han et al., 2022). Additionally, ROS can stimulate nitric oxide (NO) production via nitric oxide synthase (NOS), further damaging mitochondria and amplifying oxidative stress, ultimately leads to energy failure and cell death (Han et al., 2022).

Recent studies implicate specific ion channel complexes in this process. For instance, signaling through the NMDAR-TRPM4 complex or via NOS-mediated activation of TRPM7 channels contributes to calcium overload and organelle damage through undefined second messengers, which may also influence System Xc-expression and function (Ramírez et al., 2025).

#### 3.1.2 Nrf2 signaling pathway activation

Nuclear factor E2-related factor 2 (Nrf2) is a master regulator of antioxidant response. Under normal conditions, it binds to Kelch-like ECH-associated protein 1 (Keap 1) which leads to its ubiquitination and degradation. SLC7A11 is a target of Nrf2 and can be upregulated upon Nrf2 activation (Xu et al., 2019). Under oxidative stress induced by NMDAR activation, elevated ROS levels disrupt the Nrf2-Keap1 complex, allowing Nrf2 to translocate to the nucleus. There, it binds to antioxidant response elements (AREs) and upregulates a battery of cytoprotective genes, including *slc7a11* (Hu et al., 2022; Habib et al., 2015). Nrf2 activation significantly enhances the transcriptional activity of the *slc7a11* promoter, increasing SLC7A11 protein expression (Hu et al., 2022). This upregulation represents a compensatory, protective feedback mechanism, boosting System Xc-activity to enhance cystine import and GSH synthesis in an attempt to counteract NMDAR-driven oxidative stress (Habib et al., 2015).

#### 3.1.3 PP2A/AMPK/HMGB1 signaling axis

AMP-activated protein kinase (AMPK) is a central energy sensor, and its activation can suppress processes that favor ferroptosis, such as polyunsaturated fatty acid synthesis (Hsu et al., 2022). Recent evidence indicates that NMDAR activation can negatively regulate System Xc-thorugh the PP2A/AMPK/HMGB1 pathway. Specifically, NMDAR stimulation increases protein phosphatase 2A (PP2A) activity, leading to AMPK dephosphorylation and subsequent upregulation of High Mobility Group Box 1 (HMGB1). Elevated HMGB1 may then directly or indirectly repress *slc7a11* gene expression, resulting in decreased GPX4 levels and GSH concentration, thereby exacerbating oxidative damage (Han et al., 2024).

#### 3.1.4 Alterations in the intracellular environment

Beyond the above described pathways, NMDAR activation induces broader changes in the intracellular milieu that impact System Xc-. It can elevate labile iron ion (Fe^2+^) pools, which, via the Fenton reaction, generate hydroxyl radicals that deplete GSH and inactivate GPX4, promoting lipid peroxidation (Fan et al., 2023). The NMDAR inhibitor MK-801 can attenuate this process, restoring GSH and reducing ROS in models of ferroptosis. Furthermore, during ischemia, NMDAR overactivation not only mediates the inward flow of sodium ions (Na^+^), but may also further disrupt intracellular ion homeostasis through activation of other ion channels, such as TRPM7 (Sun, 2017). These ion fluxes lead to changes in intracellular pH, typically manifested as intracellular acidification (Rathje et al., 2013). In acidic environments, the activity of System Xc^−^ may be inhibited, which in turn reduces the efficiency of cystine/glutamate transport and decreases intracellular cystine uptake. This alteration results in diminished GSH synthesis, thereby weakening the cell’s antioxidant capacity (Wang et al., 2023).

### 3.2 Pharmacological implications

Modulating NMDARs-mediated signaling could influence System Xc-pathway function, potentially offering a strategy to target this pathway in neurological disorders (Table 2). Evidence suggests that pathological NMDAR activation can regulate SLC7A11 expression at transcriptional or post-transcriptional levels via the aforementioned signaling axes, thereby impacting the downstream GSH/GPX4 antioxidant axis (Han et al., 2024; Fan et al., 2025; Jimenez-Blasco et al., 2015; Amar et al., 2021). This indicates that the neuroprotection afforded by NMDAR blockade may, in part, stem from preserving System Xc-function (Han et al., 2024; Vlašić et al., 2025; Amar et al., 2021). While conceptually attractive, translating this strategy is challenging. The limited clinical success of broad-spectrum NMDAR antagonists (e.g., memantine) suggests that mere receptor blockade may be insufficient to restore a compromised System Xc-/GSH axis and risks disrupting physiological NMDAR signaling (Tauskela and Blondeau, 2025). The future likely lies in developing more precise interventions, such as subtype-selective NMDAR modulators or compounds that selectively disrupt pathological signaling complexes (e.g., NMDAR-TRPM4) linked to System Xc-inhibition. Such approaches aim to decouple pathological excitotoxicity from its detrimental impact on cellular redox balance, offering a potentially wider and more effective therapeutic window.

**TABLE 2:** Mechanisms and therapeutic targeting of NMDAR-mediated regulation on system Xc-.

NMDARs-mediated signaling axes	Mechanisms	Potential therapeutic strategies
NMDAR-Nrf2-SLC7A11-GPX4	- Astrocytic NMDAR activity boosts p35/Cdk5-mediated Nrf2 phosphorylation and activation that stimulates the release of GSH precursors in neurons (Jimenez-Blasco et al., 2015) - Nrf2 as a transcriptional factor increasing the expression of *slc7a11* and GPX4 (Yuan et al., 2021)	Nrf2 activators (Davies et al., 2021; Fu et al., 2022)
NMDAR-p53-SLC7A11-GSH	- p53 binds to the promoter region of *slc7a11* to repress its expression, further decreasing GSH synthesis and activating ferroptosis (Zhai et al., 2023) - NMDA-induced excitotoxicity increases levels of p53 gene and protein (Lambuk et al., 2021)	NMDA receptor blocker MK-801 (Vlašić et al., 2025)
NMDAR-PP2A-AMPK-HMGB1-GPX4	- NMDARs activation regulates endothelial ferroptosis via the PP2A-AMPK-HMGB1 axis (Han et al., 2024)	- NMDAR inhibitor MK-801 (Han et al., 2024)
NMDAR-BACH1-SLC7A11-GSH	- BACH1 promotes ferroptosis by reducing *slc7a11* expression and GSH synthesis (Nishizawa et al., 2020) - G9a interacts with BACH1 and activates ferroptosis by suppressing the transcription of Slc7a11 via dimethylation of H3K9me2 (Yao et al., 2024) - QZZG inhibits neuroexcitotoxicity and ferroptosis by regulating the NMDAR/NRF2/BACH1/ACSL4 pathway (Fan et al., 2025)	- Down-regulation of BACH1 (Nishizawa et al., 2020) - TCM: QZZG to promote neuroprotective GluN2A expression (Fan et al., 2025)
NMDAR-ATF4-SLC7A11-GSH	- Mutation of ATF4 leads to constitutive upregulation of SLC7A11 and subsequently cellular GSH (Kang et al., 2023) - cLTP-induced ATF4 downregulation is dependent on NMDAR activation (Amar et al., 2021)	NMDA receptor antagonist AP5/7CK (Amar et al., 2021)
NMDAR-BECN1-SLC7A11	- BECN1-SLC7A11 complex formation contributes to lipid peroxidation in ferroptosis (Song et al., 2018) - The autophagy protein BECN1 is activated early after NMDA stimulation (Montiel et al., 2020)	β-hydroxybutyrate (Montiel et al., 2020)

## 4 The role of system Xc-pathway in neurological disorders

System Xc-is not merely a bystander but is dynamically dysregulated, contributing to core disease mechanisms in a manner that is often cell-type and disease-context specific. The following sections will systematically analyze the distinct, and sometimes paradoxical, roles of the System Xc-pathway in five key disorders, including ischemic stroke, Alzheimer’s disease, multiple sclerosis, Parkinson’s disease, and amyotrophic lateral sclerosis. This examination will highlight how its function shifts from protective to pathogenic, thereby defining unique therapeutic challenges and opportunities in each disease (Table 3).

**TABLE 3:** The role of System Xc-pathway in neurological disorders.

Disorders	Dysregulation pattern	Mechanistic pathways	Evidence consistency	Therapeutic implications
Ischemic stroke	- Initial compensatory regulation (Soria et al., 2014) - Followed ROS-mediated inhibition and maladaptive HIF-1α-driven overactivation (Hsieh et al., 2017)	A pivotal nexus balancing adaptive antioxidant signaling (NRF2/SLC7A11) and maladaptive excitotoxic reinforcement (HIF-1α/NMDARs/ROS) (Hu et al., 2022; Hsieh et al., 2017; Sharma et al., 2016)	- Protective in moderate ischemia (PTI model) (He and Hewett, 2022) - Neutral/overwhelmed in severe ischemia (pMCAO model) (He and Hewett, 2022) - Post-translational regulation (FTO-OTUB1 axis) (Shen et al., 2025)	- Sorafenib or S-4-Carboxyphenylglycine shows efficacy in reperfusion models (Hsieh et al., 2017; Heit et al., 2024) - Combination strategy with recanalization therapies (e.g., tPA) (Hsieh et al., 2017; Heit et al., 2024) - Staged therapeutic window: inhibition in the acute excitotoxic phase, with potential support during recovery (Li et al., 2023)
Alzheimer’s disease	Cell specific dichotomy - Impaired in neurons (Braidy et al., 2019; Ayala et al., 2014) - Upregulated in reactive astrocytes (Ashraf et al., 2020; Schallier et al., 2011)	Neurons: reducing cystine uptake/GSH to promote ferroptosis (Braidy et al., 2019; Ayala et al., 2014) Astrocytes: driving excessive glutamate release to exacerbate excitotoxicity (Ashraf et al., 2020; Schallier et al., 2011)	A dual therapeutic logic Enhancing System Xc- in neurons while inhibiting it in astrocytes is beneficial (Dahlmanns et al., 2023)	- Neuron-targeted activation (e.g., NRF2 agonists like GAA) (Lu et al., 2025) - Astrocyte-targeted inhibition (e.g., Sulfasalazine) (D'Ezio et al., 2021) - Indirect astrocyte modulation (e.g., Lactoferrin overexpression) (Fan et al., 2024)
Multiple sclerosis	- Pathogenic driver in neuroimmune axis (Evonuk et al., 2015; Domercq et al., 2007; Rosin et al., 2004) - Cytoprotective factor in oligodendrocytes (Ambrosius et al., 2020; Vivinetto et al., 2022)	- Facilitate CNS infiltration of T cells (Evonuk et al., 2015) - Drive excitotoxic glutamate release in activated glia (Domercq et al., 2007; Rosin et al., 2004) - Antioxidant defense via MAG-NRF2 axis and glutamate clearance for myelin integrity in oligodendrocytes (Ambrosius et al., 2020; Vivinetto et al., 2022)	- Inhibition beneficial in EAE model (e.g., Sulfasalazine) (Evonuk et al., 2015) - Broad protective pathway activation failed in clinical trials (e.g., anti-MAG therapy) (Cramer et al., 2017)	- Inhibit in neuroimmune axis (e.g., Sulfasalazine) (Evonuk et al., 2015) - Preserve/activate in oligodendrocytes (e.g., via NRF2) (Cramer et al., 2017) - Develop advanced cell-selective delivery systems
Parkinson’s disease	A central node in oxidative stress and neuroinflammation (Gao et al., 2025)	- Core vicious cycle: α-syn misfolding-System Xc- inhibition-Oxidative stress/ferroptosis-α-syn aggregation (Angelova et al., 2021) - LRRK2 upregulation engages System Xc-/GSH/GPX4 axis to drive microglial ferroptosis and inflammation (Zheng et al., 2024; Xiong and Yu, 2024)	- LRRK2 inhibition is protective in cellular/MPTP models (Zheng et al., 2024) - Limited clinical translatability for direct GSH replenishment (Mischley et al., 2017; Sechi et al., 1996)	- Upstream activation: using GSH precursor NAC (Asanuma and Miyazaki, 2021) - Target the vicious cycle - Modulate neuroinflammation: LRRK2 inhibition (e.g., PF-06447475) (Zheng et al., 2024)
Amyotrophic lateral sclerosis	A critical integrator of oxidative stress, neuroinflammation, and excitotoxicity (Gao et al., 2023)	- Oxidative stress: impaired System Xc-/GSH/GPX4 axis (Gao et al., 2023) - Neuroinflammation: microglial shift to a pro-inflammatory phenotype (Gao et al., 2023) - A vicious cycle of NMDAR-mediated excitotoxicity: glial glutamate release mediated by System Xc- and impaired EAAT2 uptake (Pajarillo et al., 2019; Albano et al., 2013)	*slc7a11* knockout attenuates disease in SOD1 mice (Guo et al., 2024)	- Riluzole (glutamate release inhibitor/NMDAR antagonist) (Cheah et al., 2010; Can et al., 2017) - Edaravone (free radical scavenger/GPX4 stabilizer) (Tzeplaeff et al., 2023) - Edaravone Dexboeol (EDB) (combinatorial, NMDAR-modulating) (Chen et al., 2022; Guo et al., 2024; Qiu et al., 2025)

### 4.1 System Xc-in ischemic stroke: The paradox of dual roles

The dysfunction of System Xc-following cerebral ischemia is complex. During the initial phase, overactivation of NMDARs generates ROS, which can trigger a compensatory upregulation of SLC7A11 in attempt to enhance GSH synthesis (Han et al., 2024; Soria et al., 2014). Paradoxically, sustained high ROS levels may oxidize and inhibit the SLC7A11 protein itself, crippling this defense mechanism and leading to GSH depletion (Shi et al., 2023; Fan et al., 2023; Han et al., 2022). Concurrently, hypoxia-inducible factor-1α (HIF-1α) can directly upregulate SLC7A11 transcription. This may lead to a maladaptive response: increased System Xc-activity drives excessive glutamate release, which further fuels NMDAR overactivation and excitotoxicity, creating a vicious cycle that exacerbates ischemic damage (Fan et al., 2023; Hsieh et al., 2017). HIF-1α can mediate increased sympathoexcitation via up-regulating NMDARs in rodents with chronic heart failure (Sharma et al., 2016). Whether HIF-1α directly bridges this process by regulating NMDARs remains an open question. Mechanistic pathways thus involve a precarious balance between adaptive antioxidant upregulation (via NRF2/SLC7A11) and maladaptive excitotoxic reinforcement (via HIF-1α/NMDARs/ROS), with the cellular redox state determining the functional outcome of System Xc-activity (Hu et al., 2022; Hsieh et al., 2017; Sharma et al., 2016).

Experimental evidence also directly reflects this mechanistic duality. Genetic knockout of xCT was protective in the milder photothrombotic ischemia (PTI) model but showed no benefit in the more severe permanent middle cerebral artery occlusion (pMCAO) model (He and Hewett, 2022). This suggests that the protective role of System Xc-may be overwhelmed in profound ischemia. Furthermore, post-translational regulation is significant, as enhancing SLC7A11 stability (via the FTO-OTUB1 axis) can reduce ferroptosis in reperfusion models (Shen et al., 2025). These findings collectively paint a picture of a dual-role System Xc-: its moderate activity is crucial for redox balance, while its excessive or dysfunctional activity exacerbates injury (Supplementary Figure S1). Given this paradox, therapeutic implications are nuanced and must consider timing and ischemic status. Pharmacological inhibition (e.g., with sorafenib or S-4-carboxyphenylglycine) has shown promise in reducing infarct volume and extending the treatment window in reperfusion models, potentially by dampening excitotoxicity in the penumbra (Hsieh et al., 2017; Heit et al., 2024). A promising strategic approach may involve combining System Xc-inhibitors with recanalization therapies like tissue plasminogen activator (tPA), aiming to counteract excitotoxic damage while restoring blood flow (Li et al., 2023; Hsieh et al., 2017; Heit et al., 2024). Future interventions may need to be selectively deployed-potentially inhibiting System Xc-in the acute excitotoxic phase while supporting its function during recovery.

### 4.2 System Xc-in Alzheimer’s disease: A cell-specific regulator

Alzheimer’s disease (AD) is characterized by the accumulation of amyloid-β (Aβ) plaques and neurofibrillary tangles (Serrano-Pozo et al., 2011; Zhang et al., 2023). Elevated brain iron levels in AD patients, closely associated with Aβ and tau pathology, strongly implicate ferroptosis in disease progression (Zhang et al., 2022). Within this context, System Xc-serves as a critical node, regulating ferroptosis, glutamate excitotoxicity, and oxidative stress in a cell-type-specific manner (Supplementary Figure S2). In neurons, Aβ oligomers induce oxidative stress that impairs System Xc-function, reducing cystine uptake and GSH synthesis. This, coupled with decreased GPX4 expression, leads to the accumulation of toxic lipid peroxidation products (e.g., 4-HNE, MDA), increasing neuronal vulnerability to ferroptotic death (Braidy et al., 2019; Ayala et al., 2014). Consequently, enhancing neuronal System Xc-activity via the NRF2 pathway (e.g., with Ganoderic acid A) improves cognitive deficits in AD models by upregulating SLC7A11/GPX4 and reducing oxidative damage (Lu et al., 2025). Conversely, in reactive astrocytes, System Xc-is often upregulated. *In vitro*, Aβ peptides stimulate astrocytic System Xc-, leading to excessive glutamate release and neuronal death - an effect preventable by the inhibitor sulfasalazine (Ashraf et al., 2020; Schallier et al., 2011; D'Ezio et al., 2021). This suggests that selectively inhibiting astrocytic System Xc-could be therapeutic without compromising neuronal antioxidant defenses (Dahlmanns et al., 2023). An alternative protective strategy involves modulating astrocytic function indirectly. For example, overexpressing astrocytic lactoferrin reduces neuronal iron accumulation and prevents GPX4 degradation, thereby inhibiting ferroptosis (Fan et al., 2024). In summary, System Xc-in AD presents a dual profile: its impairment in neurons exacerbates oxidative stress and ferroptosis, while its upregulation in reactive astrocytes may drive excitotoxicity. This cell-specific duality defines a central challenge and opportunity for therapy, underscoring the need for targeted strategies that either restore neuronal antioxidant capacity or normalize astrocytic glutamate release.

### 4.3 System Xc-in multiple sclerosis: a double-edged sword in neuroinflammation and myelin integrity

Multiple Sclerosis (MS) is a chronic, immune-mediated demyelinating disorder of the CNS (Aliyu et al., 2024; Sumida et al., 2024). Within its complex pathogenesis, System Xc-emerges as a critical but multifaceted player, primarily recognized for regulating immune cell infiltration. In the experimental autoimmune encephalomyelitis (EAE) model, pharmacological inhibition of System Xc-with sulfasalazine or its genetic deletion significantly reduces CNS infiltration of T cells, ameliorates clinical symptoms, and limits demyelination, without impairing peripheral T-cell activation (Evonuk et al., 2015). This underscores its specific role in facilitating T-cell migration across the CNS barrier.

Beyond immune trafficking, System Xc-dysfunction in activated microglia and astrocytes contributes to the neuroinflammatory milieu. Aberrant activity in these cells can lead to excessive glutamate release, which exacerbates disease progression through dual pathways: over-activation of NMDARs causing excitotoxicity, and induction of oxidative stress that is toxic to oligodendrocytes (Domercq et al., 2007; Rosin et al., 2004). Conversely, within oligodendrocytes as the myelin-forming cells, System Xc-serves a vital protective function. It is integral to the antioxidant defense necessary for myelin maintenance. Notably, enhancing System Xc-expression specifically in oligodendrocytes, for example via the myelin-associated glycoprotein (MAG)-NRF2 axis, can upregulate SLC7A11 and promote extracellular glutamate clearance, demonstrating a neuroprotective effect against excitotoxicity (Ambrosius et al., 2020; Vivinetto et al., 2022). However, the failure of broader anti-MAG therapy in clinical trials highlights the challenge of translating specific protective pathways into effective systemic treatments (Cramer et al., 2017). Taken together, System Xc-in MS exhibits a cell-type-dependent duality: it acts as a pathogenic driver in the neuroimmune axis (promoting infiltration and glial-mediated excitotoxicity) and as a cytoprotective factor in oligodendrocytes (Supplementary Figure S3). This inherent complexity creates a significant therapeutic challenge, emphasizing the need for future strategies that can achieve cell-specific modulation of this transporter to selectively inhibit its detrimental roles while preserving its essential protective functions.

### 4.4 System Xc-in Parkinson’s disease: a central node in oxidative stress and neuroinflammation

Parkinson’s Disease (PD) is characterized by the progressive loss of dopaminergic neurons in the substantia nigra, accompanied by neuroinflammation and the accumulation of α-synuclein (α-syn) (Kalia and Lang, 2015). System Xc-dysfunction is a key contributor to PD pathogenesis, primarily by exacerbating oxidative stress and ferroptosis (Gao et al., 2025). A vicious cycle links these processes: α-syn misfolding can disrupt lysosomal function, further inhibiting System Xc-activity, which in turn amplifies oxidative stress and promotes more α-syn aggregation (Angelova et al., 2021). This creates a self-reinforcing loop of “oxidative stress-ferroptosis-protein aggregation” that drives neurodegeneration (Supplementary Figure S4).

The role of System Xc-also extends into neuroinflammation. The expression of leucine-rich repeat protein kinase 2 (LRRK2) is upregulated in PD, which critically intersects with System Xc-pathway. The System Xc-/GSH/GPX4 axis has been identified as a major effector of LRRK2-mediated microglial ferroptosis and inflammatory activation (Zheng et al., 2024; Xiong and Yu, 2024). Inhibiting LRRK2 in lipopolysaccharide-treated cellular model or 1-methyl-4-phenyl-1,2,3,6-tetrahydropyridine (MPTP)-induced animal models (e.g., with PF-06447475) suppresses pro-inflammatory cytokine release, promotes neuroprotective factor secretion, and prevents neuronal apoptosis, highlighting this node as a therapeutic target (Zheng et al., 2024). GSH depletion is an early and critical event in nigral degeneration, leading to mitochondrial damage via ROS (Tardiolo et al., 2018). While directly replenishing GSH showed promise in a clinical trial by improving the Unified PD Rating Scale (UPDRS) scores, its therapeutic application is hindered by pharmacokinetic challenges, including poor membrane permeability and a short half-life (Mischley et al., 2017; Sechi et al., 1996). Therefore, strategies focused on upstream activation of System Xc-to boost endogenous GSH synthesis are compelling. As a precursor for the production of GSH, N-acetylcysteine (NAC), which can cross the blood-brain barrier, activate System Xc-, and protect mitochondria, represents one such promising approach (Asanuma and Miyazaki, 2021).

### 4.5 System Xc-in amyotrophic lateral sclerosis: integrating oxidative stress, neuroinflammation and excitotoxicity

Amyotrophic Lateral Sclerosis (ALS) is a fatal neurodegenerative disease involving the progressive loss of upper and lower motor neurons (Feldman et al., 2022). A central feature of ALS pathogenesis is significant oxidative stress, marked by elevated ROS and diminished antioxidant capacity in the CNS (Pinilla-González et al., 2024). This redox imbalance is closely linked to functional impairment of the System Xc-/GSH/GPX4 axis, leading to GSH depletion and increased susceptibility to ferroptosis. Concurrently, System Xc-dysregulation in microglia promotes a shift to a pro-inflammatory phenotype, releasing cytotoxic cytokines (e.g., TNF-α, IL-1β) (Gao et al., 2023). Furthermore, elevated extracellular glutamate, resulting from System Xc-mediated release by activated glia combined with impaired EAAT2-dependent uptake, fuels NMDAR-mediated excitotoxicity. This creates a vicious cycle that exacerbates neuronal injury (Pajarillo et al., 2019; Albano et al., 2013). Notably, genetic knockout of *slc7a11* in SOD1 mutant mice attenuates disease progression, suggesting that chronic, pathological upregulation of System Xc-may be a net contributor to toxicity, likely through exacerbating excitotoxicity (Mesci et al., 2015). This paradox underscores the complexity of targeting this pathway (Supplementary Figure S5).

This interplay informs the mechanism of existing and potential therapies. The approved drugs riluzole (an inhibitor of glutamate release and NMDAR antagonist) and edaravone (a free radical scavenger that may stabilize GPX4) indirectly target pathways intertwined with System Xc-dysfunction (Cheah et al., 2010; Can et al., 2017; Tzeplaeff et al., 2023; Martinez et al., 2023). Their combinatorial formulation, edaravone dexboeol (EDB), shows enhanced neuroprotection, potentially through modulating NMDAR signaling (Chen et al., 2022; Guo et al., 2024; Qiu et al., 2025). Whether these benefits involve normalizing System Xc-function remains a key question, highlighting System Xc-as a critical integrative node for future therapeutic strategies.

## 5 Future perspectives and challenges

### 5.1 Evolving the pharmacological toolbox

Current System Xc-modulators have significant limitations. Sulfasalazine has poor CNS penetration and potent anti-inflammatory off-target effects (Verbruggen et al., 2021). Erastin and its analogs are non-selective and metabolically unstable, and Sorafenib is a multi-kinase inhibitor with a vast profile of actions (Stockwell and Jiang, 2020). These limitations necessitate cautious interpretation of *in vivo* phenotypes attributed solely to System Xc-inhibition. Future efforts must prioritize the development of novel, brain-penetrant compounds with higher selectivity and improved drug-like properties, potentially guided by emerging structural insights into SLC7A11.

### 5.2 Towards nuanced targeting strategies

Considering that the role of System Xc-is context-dependent or varies with different diseases, future therapeutic strategies must be sophisticated. i) Temporal and spatial precision. Short-term inhibition may be warranted in acute excitotoxic phases (e.g., ischemic stroke), while chronic neurodegenerative conditions may require enhancing function in vulnerable neurons or selectively inhibiting it in pathogenic glia. ii) Rational combination therapies. System Xc-modulators could be combined with NMDAR-targeting drugs, anti-inflammatory agents, or iron chelators to achieve synergistic efficacy and broader therapeutic windows (Sahin et al., 2025). iii) Upstream epigenetic and transcriptional modulation. Targeting regulators of SLC7A11 expression (e.g., Nrf2 activators, Bach1 inhibitors, specific miRNAs) may offer a more physiological means of tuning its activity.

### 5.3 Critical knowledge gaps and potential research directions

To define cell-Specific mechanisms, employing conditional genetic tools in disease-relevant models is imperative to dissect the precise contribution of System Xc-in different nerve cells. Given its non-ferroptotic Functions, the impact of System Xc-modulation on neuroimmune signaling and brain metabolism should be factored into therapeutic development and safety assessments. Moreover, identifying imaging or fluid biomarkers for System Xc-activity, ferroptosis, and excitotoxicity is crucial for patient stratification and treatment monitoring in clinical trials. To address the BBB penetration challenge, advanced delivery systems (nanocarriers, focused ultrasound-mediated BBB opening) should be integrated into the preclinical development pipeline.

## 6 Conclusion

In conclusion, the System Xc-pathway emerges not as a simple substrate transporter but as a critical CNS hub integrating redox balance, glutamate signaling, metabolic coupling, and immune function. Its bidirectional crosstalk with NMDARs forms a core amplifier loop for neural damage. Its role in neurological diseases is fundamentally context-dependent and often paradoxical, which is a direct consequence of its diverse cellular distribution and multifunctional nature. The future of neuropharmacology targeting this pathway must abandon a “one-size-fits-all” approach. Success will depend on developing temporally precise, cell-type-specific, and potentially combinatorial strategies. Overcoming the limitations of current pharmacological tools and deepening our understanding of its complex biology within the intact neurovascular unit are essential steps to translate System Xc-from a compelling molecular target into effective therapies for a range of devastating neurological disorders.
