# Supplementary material for: System Xc-pathway as a potential regulatory target in neurological disorders
Source: Front Pharmacol. 2026 Jan 2;16:1701320. doi: 10.3389/fphar.2025.1701320 (PMC12808454; doi:10.3389/fphar.2025.1701320)
Supplement: Supplementary file 1 [file DataSheet1.doc]

**Supplementary Materials**


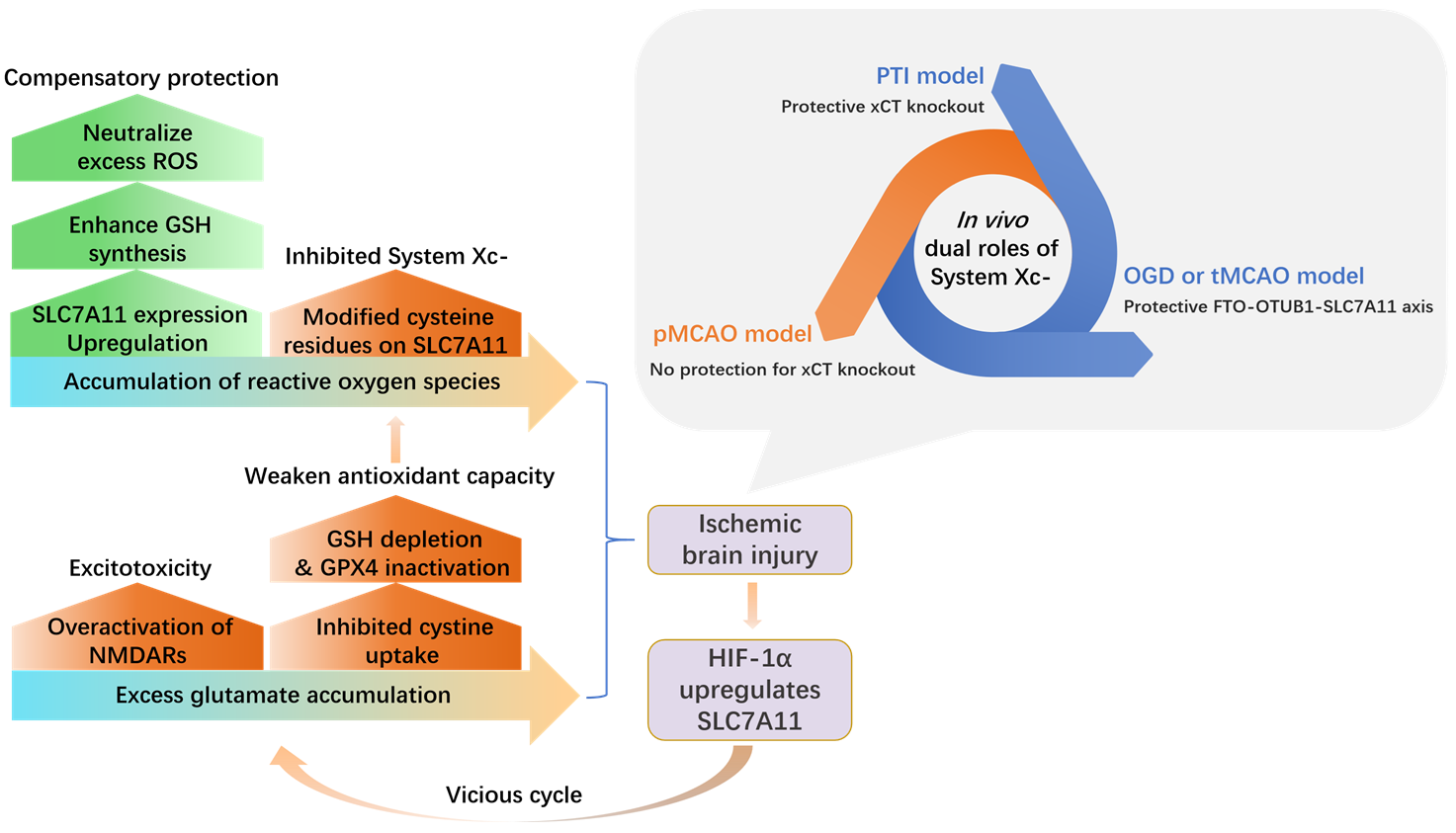


**Supplementary Figure S1. Dual roles of System Xc- exhibited in mechanisms and models of ischemic stroke.** Moderate activation of System Xc- may exert protective effects by regulating intracellular redox balance. Nevertheless, excessive activation of System Xc- may lead to increased glutamate release and weaken antioxidant capacity of oxidative stress, ultimately exacerbating ischemic injury. *In vivo* animal experiments have also demonstrated that whether the effect of System Xc- is protective or injurious depends on the different models and the degree of severity.


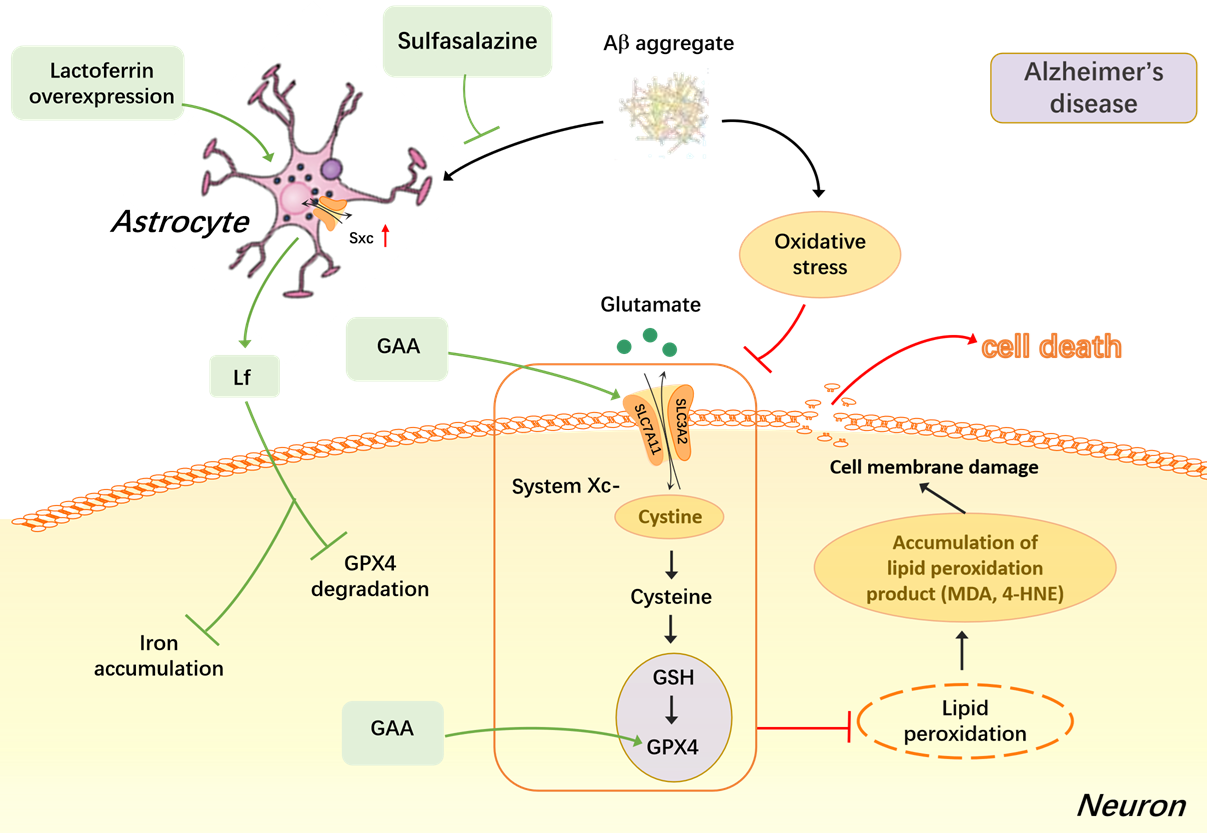


**Supplementary Figure S2. The distinct roles of neuronal and astrocytic System Xc- in Alzheimer’s disease (AD).** Amyloid-β (Aβ) oligomers may induce oxidative stress, which in turn inhibit the function of neuronal System Xc-/GSH/GPX4 axis, leading to the accumulation of lipid peroxidation products and ultimately resulting in neuronal death. Ganoderic acid A (GAA) may improve AD-related cognitive impairment by promoting the function of neuronal System Xc-. Conversely, Aβ-induced upregulation of System Xc- in astrocytes may lead to neuronal cell death, which can be prevented by the System Xc- inhibitor Sulfasalazine (SAS). Moreover, overexpression of lactoferrin (Lf) in astrocytes can inhibit neuronal ferroptosis by reducing intracellular iron accumulation and preventing GPX4 degradation.


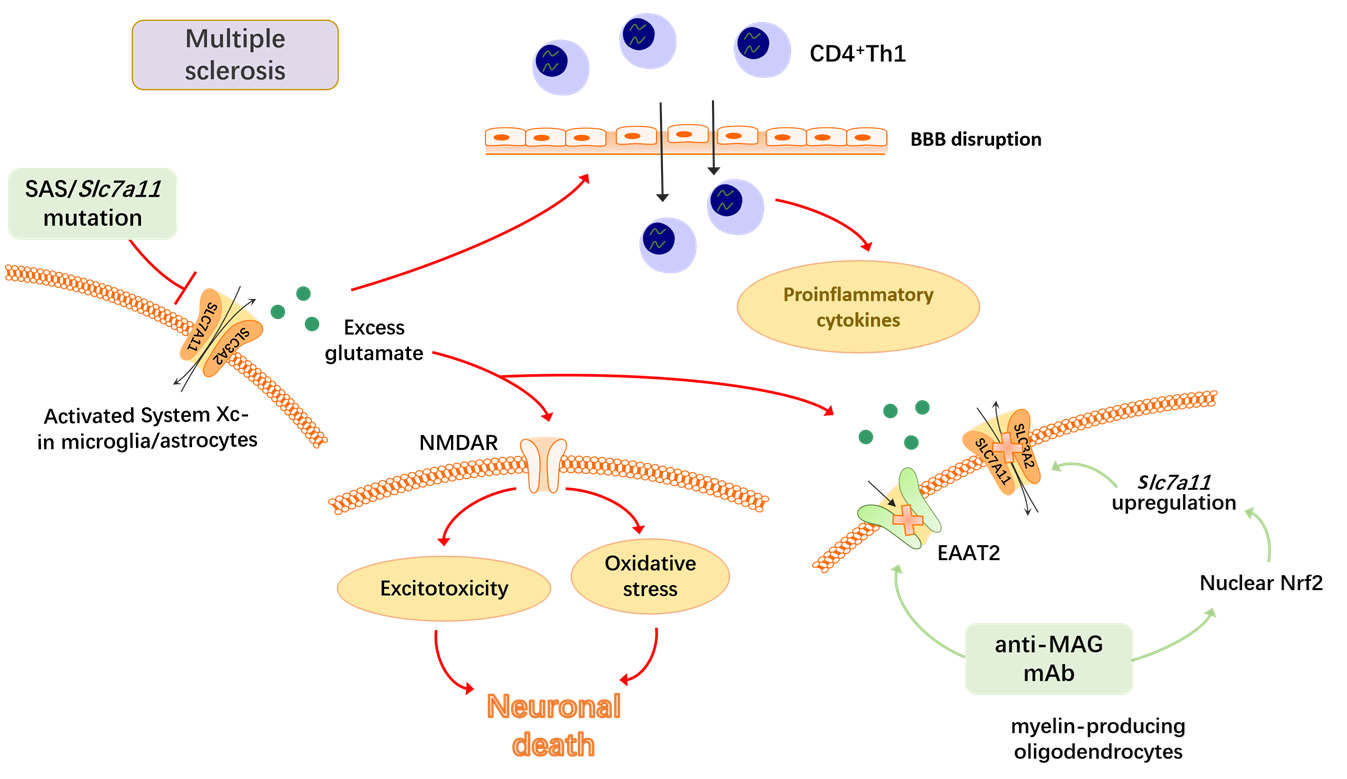


**Supplementary Figure S3. The central role of System Xc- in autoimmune inflammatory demyelinating diseases.** In multiple sclerosis (MS), the activation of microglia and astrocytes can disrupt the normal function of System Xc-. This dysfunction facilitates the infiltration of immune cells into the CNS through a compromised blood-brain barrier (BBB), thereby triggering inflammatory responses. Concurrently, it leads to excess accumulation of glutamate, which further exacerbates oxidative stress and excitotoxicity in myelin-producing oligodendrocytes, ultimately causing cell death. Study in experimental autoimmune encephalomyelitis (EAE) have demonstrated that inhibiting System Xc- using sulfasalazine (SAS) or inducing a genetic mutation in *Slc7a11*, as well as anti-MAG therapy, may mitigate disease progression, highlighting the protective potential of targeting System Xc- in specific cell types.


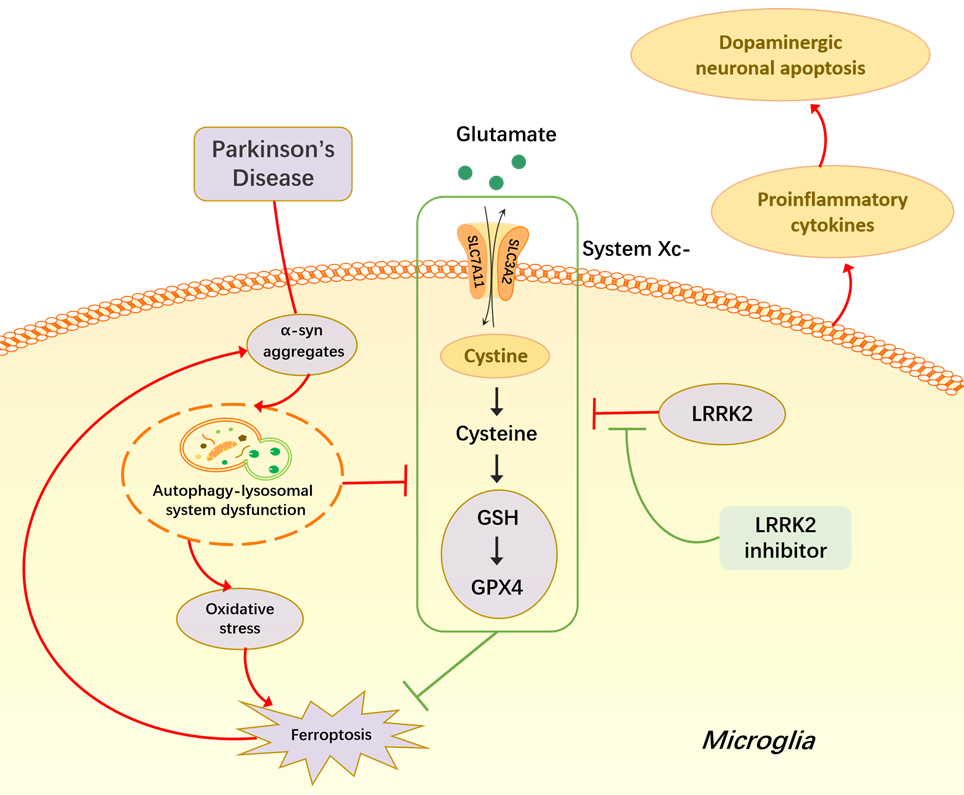


**Supplementary Figure S4. The pivotal role of microglial System Xc- in the pathogenesis of Parkinson’s disease (PD).** System Xc- and ferroptosis pathways are integral to PD pathogenesis, interacting with α-syn to create a detrimental cycle in microglia. Inhibition of Leucine-rich repeat protein kinase 2 (LRRK2) has demonstrated potential in mitigating neuroinflammation and neuronal apoptosis in PD models.


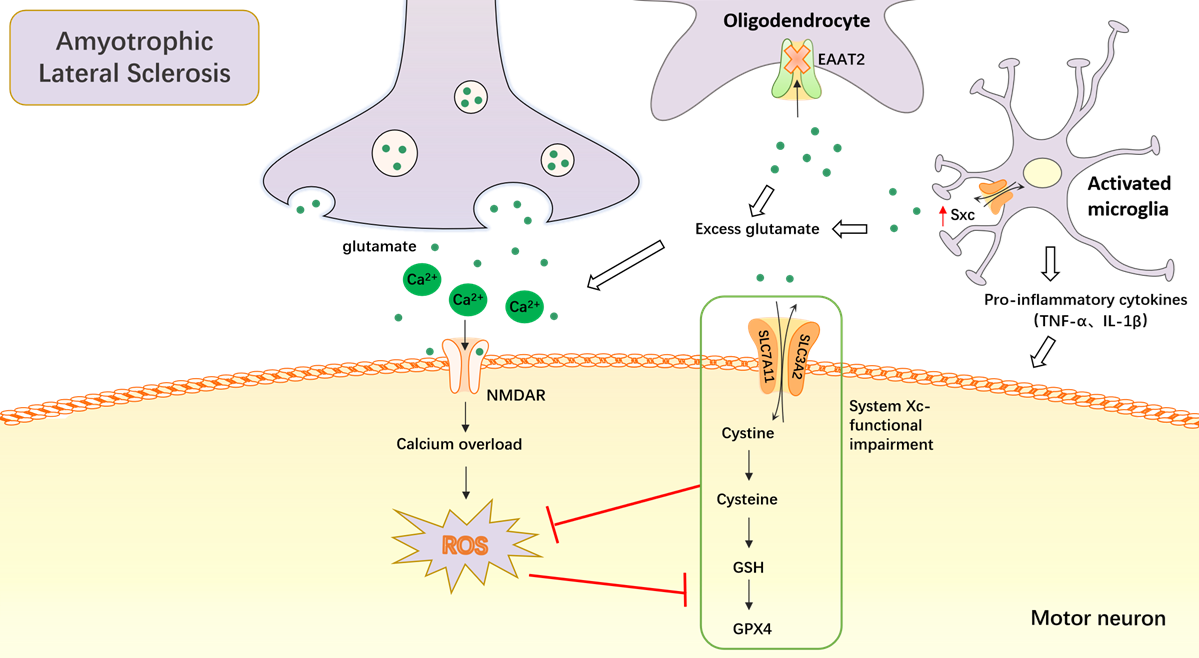


**Supplementary Figure S5. The potential role of System Xc- in the pathogenesis of Amyotrophic lateral sclerosis (ALS).** ALS is a fatal neurodegenerative disease characterized by progressive degeneration of motor neurons, ultimately resulting in muscle atrophy and respiratory failure. Oxidative stress and glutamate excitotoxicity are central to ALS pathogenesis. System Xc- (Sxc) plays a pivotal role by downregulating the glial excitatory amino acid transporter 2 (EAAT2), regulating glutamate release, modulating oxidative stress, and influencing neuroinflammation. Elevated or impaired System Xc- activity in ALS models exacerbates neuronal damage. Targeting System Xc- may thus represent a promising therapeutic strategy for ALS.
